# Quantitative Proteomic Profiling of Small Molecule Treated Mesenchymal Stem Cells Using Chemical Probes

**DOI:** 10.3390/ijms22010160

**Published:** 2020-12-26

**Authors:** Jerran Santos, Sibasish Dolai, Matthew B. O’Rourke, Fei Liu, Matthew P. Padula, Mark P. Molloy, Bruce K. Milthorpe

**Affiliations:** 1 Advanced Tissue Engineering and Stem Cell Biology Group, School of Life Sciences, University of Technology Sydney, P.O. Box 123, Broadway, NSW 2007, Australia; Bruce.Milthorpe@uts.edu.au; 2 School of Life Sciences, Faculty of Science, University of Technology Sydney, P.O. Box 123, Broadway, NSW 2007, Australia; Matthew.Padula@uts.edu.au; 3 Department of Chemistry and Biomolecular Sciences, Macquarie University, North Ryde, NSW 2109, Australia; sibadolai@gmail.com (S.D.); Fei.Liu@mq.edu.au (F.L.); m.molloy@sydney.edu.au (M.P.M.); 4 Northern Clinical School, Bowel Cancer & Biomarker Lab, Faculty of Medicine and Health, The University of Sydney, Lvl 8, Kolling Instiute, Royal North Shore Hospital, St. Leonards, NSW 2065, Australia; matthew.orourke@sydney.edu.au; 5 Proteomics Core Facility, Faculty of Science, University of Technology Sydney, P.O. Box 123, Broadway, NSW 2007, Australia

**Keywords:** adipose derived stem/stromal cells, small molecules, neural differentiation, quantitative proteomics, Bis-probe

## Abstract

The differentiation of human adipose derived stem cells toward a neural phenotype by small molecules has been a vogue topic in the last decade. The characterization of the produced cells has been explored on a broad scale, examining morphological and specific surface protein markers; however, the lack of insight into the expression of functional proteins and their interactive partners is required to further understand the extent of the process. The phenotypic characterization by proteomic profiling allows for a substantial in-depth analysis of the molecular machinery induced and directing the cellular changes through the process. Herein we describe the temporal analysis and quantitative profiling of neural differentiating human adipose-derived stem cells after sub-proteome enrichment using a bisindolylmaleimide chemical probe. The results show that proteins enriched by the Bis-probe were identified reproducibly with 133, 118, 126 and 89 proteins identified at timepoints 0, 1, 6 and 12, respectively. Each temporal timepoint presented several shared and unique proteins relative to neural differentiation and their interactivity. The major protein classes enriched and quantified were enzymes, structural and ribosomal proteins that are integral to differentiation pathways. There were 42 uniquely identified enzymes identified in the cells, many acting as hubs in the networks with several interactions across the network modulating key biological pathways. From the cohort, it was found by gene ontology analysis that 18 enzymes had direct involvement with neurogenic differentiation.

## 1. Introduction

Mesenchymal stem cells (MSCs) are multipotent adult cells that have an innate potential for self-renewal and the ability to divide and create a specialized cell that is phenotypically different from the parent. In the last decade, adipose tissue has been identified as a major source of MSCs as it contains a stromal vascular fraction (SVF), akin to bone marrow. The SVF is rich in adult mesenchymal stem cells, termed adipose-derived stem/stromal cells (ADSCs), in numbers a thousand-fold greater than that derived from bone marrow. In recent years, adult stem cells have been successfully used to treat joint and muscle injuries in humans and animals [1,2,3,4]; however, regenerative therapies have yet to be developed for organs such as the brain and central nervous system. These stem cell therapies have the potential to revolutionise human healthcare and medicine.

Numerous studies show that ADSCs, as well as other adult MSCs, can be induced toward a neural differentiated population in vitro [5,6,7,8] and various research groups have adopted one of two main strategies. The first, by Woodbury et al. [5], applied simple chemical compounds as media additives to induce differentiation. The second method, by Sanchez-Ramos et al., [9], utilises cytokine and growth factor media additives. Interestingly the two methods vary considerably for the time the MSCs need to spend in culture before showing the phenotypic changes that indicate differentiation. The Woodbury method only takes hours for more than 75% of the MSCs to adopt neural morphology as well as express neural specific markers, such as NeuN, (Microtubule-associated protein 2) MAP-2 and Tubulin III. Conversely, the Sanchez-Ramos method takes several weeks for the MSCs to express the same neural specific markers indicative of differentiation and with a significantly reduced percentage of total cells differentiated. Barnabé et al. [10] have shown that the Woodbury method cells are non-functional, while other studies indicate the cells are at either the pre-neural stage or are immature neuroblasts that can be further differentiated into glial cells, oligodendricytes, or astrocytes including myelin sheath forming Schwann cells that are functional [11,12,13,14,15,16]. In recent studies, b-mercaptoethanol (BME) has been used as a media constituent for the neural differentiation of embryonic stem cells, induced pluripotent stem cells (iPSC), neural stem cells and ADSCs [17,18,19,20]. Previous work by our group [8] showed that BME treated ADSCs are in a pre-neural state, presenting several neural specific markers for functionality and cellular structural rearrangement within short interval treatments. It was also observed that longer treatment time points tend to produce cells that are visually morphological phenocopies; however, they expressed stress related proteins. It was observed that several proteins detected, at the shorter time intervals that held significant biological value toward neural differentiation, were enzymes [11].

In light of the findings of the above studies, a targeted method to capture these enzymes for analysis was required to quantify and validate their abundance and roles. We have previously described a Bis-probe as a chemical reagent designed to capture enzymes which utilize purines as co-factors (e.g., kinases, NAD-binders) as these important protein classes might otherwise be difficult to detect in proteomic analyses [21]. As such the aim was to use the synthetic chemical probes to selectively capture and enrich these enzymes from these differentiating cells to enable their characterization by mass spectrometry for bioinformatic interrogation into their roles during the differentiation process.

## 2. Results

### 2.1. Neurogenic Differentiation of hADSCs

**Figure 1:**
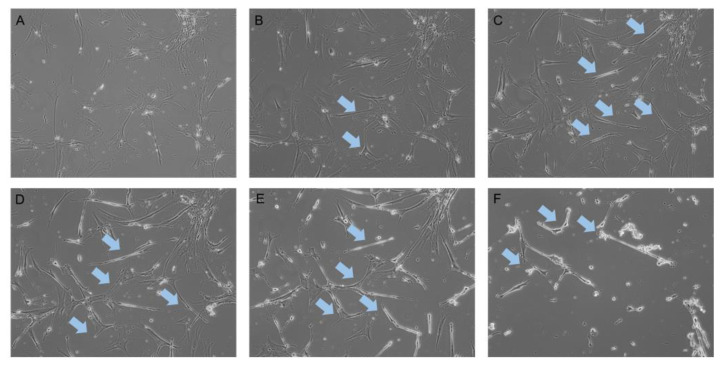
BME treated mesenchymal stem cells (MSCs) at 10× magnification (**A**) Basal ADSCs. Pre-induction media ADSCs (**B**) 6 h (**C**) 24 h. Induction Media ADSCs (**D**) 1 h (**E**) 6 h and (**F**) 12 h. The temporal progression of the treatment shows the ADSCs morphological change from flat dense fibroblastic cells to narrow, spindle-like cells with bipolar extension presenting early neurite outgrowth shown by blue arrows.

Cellular morphology is an important factor to consider in numerous contexts. In vitro culture morphology specifies the status of the cells in terms of the health of the cells, and the stage differentiation is critical. The cells exhibited in Figure 1A are healthy ADSCs in a basal non-perturbed state. Their cellular morphology is limited to irregular fibroblastic in a semi-confluent culture. Cultures for differentiation were placed in a pre-induction media (PIM) of 1 mM beta-mercaptoethanol, one-tenth the strength of the differentiation media concentration. Cellular morphology after 6 h in the PIM is relatively stable with cells exhibiting the basal fibroblastic morphology (Figure 1B). At the 24 h time point, considerable changes were apparent throughout the culture with significant cellular remodelling observable (Figure 1C). The cellular remodelling most notably affects the membrane, which retracts, giving the cells a narrower appearance. The culture appears less confluent with larger area between cells due to the decreased surface area of each cell. The cell population numbers remain relatively unchanged (data not shown).

Following the pre-induction stage, differentiation media were placed on the cells and, an hour after being exposed to the differentiation media, additional morphological changes are noticeable (Figure 1D). The cell membranes exhibit further retraction with the majority of cells forming a spindle-like bipolar structure. Within 6 h of differentiation, the morphology of the total cell population is quite distinct compared to the ADSCs in Figure 1A. The spindle-like cells have dense and highly refractive nuclei and multiple neurite extensions reaching between cells shown by blue arrows. At 6 h, the cells all appear neural-like in morphology and greatly resemble primary neurites or astrocytic cells (Figure 1E). Cell neurite extensions were observed, linking multiple cells in succession, shown by blue arrows. By 12 h (Figure 1F), the remaining attached cells present neurite links and extension between cells as well as larger cellular fragments from detachment.

### 2.2. Bis-Probe Proteome Characterization

The combination of Bis-probe enrichment along with shotgun proteomics and spectral counting using normalised spectral abundance factors enabled us to examine the changes in protein expression patterns due to the effect of BME on hADSCs.

**Figure 2:**
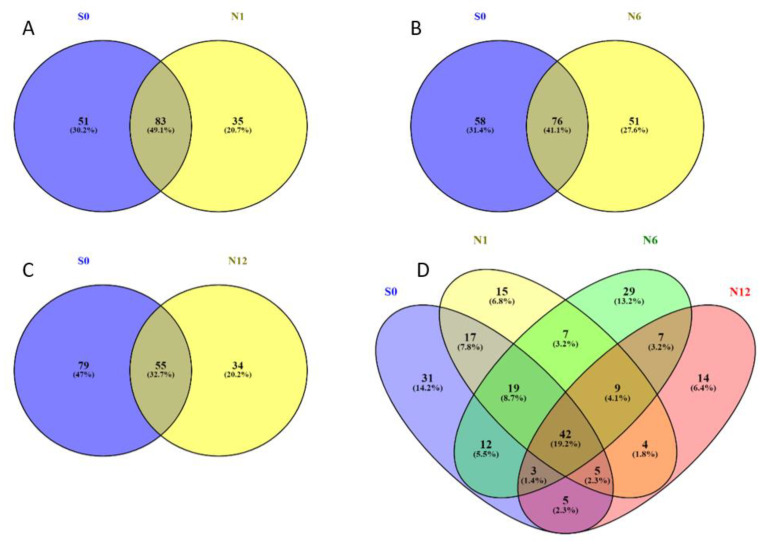
Relative quantitative shared and unique proteins captured by Bis-probe in S0, N1, N6 and N12 samples. Presented as a 2-way Venn diagram in (**A**–**C**) and a 4-way Venn diagram in (**D**).

The number of proteins enriched by the Bis-probe, and identified reproducibly in at least two of the three biological replicates, were 133 in group S0, 118 in group N1, 126 in group N6 and 89 in group N12. A large number of common and unique proteins were captured by the Bis-probe from the different treatment groups (Table 1 and Figure 2). In comparison to the control hADSCs (S0), the BME treated cells for 1 h, 6 h and 12 h had 83, 76 and 55 proteins in common and 51, 58 and 55 proteins unique to each time point, respectively (Figure 2A–C). The proteins captured by the Bis- probe were further partitioned into categories based on their presence and/or absence in each of the time points of BME treatment. In total, 42 proteins were identified that were common to all the time points, including the control group. The S0 group had 31 proteins, the N1 group had 15 proteins, the N6 group had 29 proteins and the N12 group had 14 proteins that were unique in the entire data set (Figure 2D).

**Figure 3:**
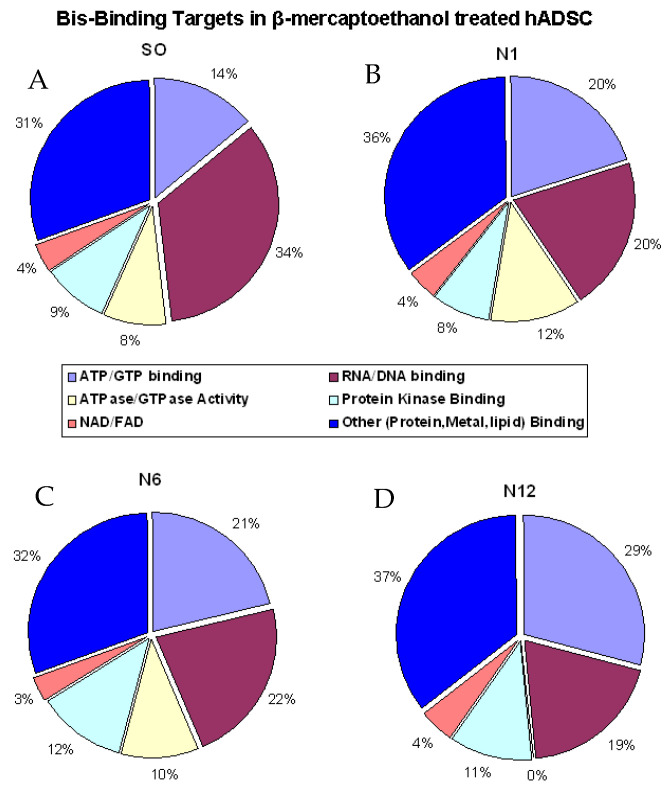

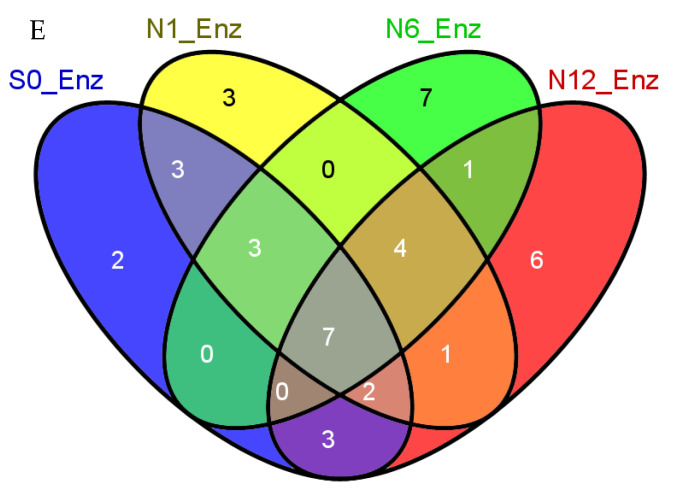
Bis-probe bound proteins in group clusters percentages in each sample treatment. (**A**) S0, (**B**) N1, (**C**) N6 and (**D**) N12. **(E)** Number of enzymes captured by the Bis-group in each treatment group.

The identified proteins in each treatment were grouped using Gene Ontology (GO) annotation and categorised based on molecular function (Figure 3). In the S0 group, approximately 70% of the Bis-probe bound proteins had reported purine binding functions and 30 % had either protein/lipid or metal binding functions or were categorised as others (Figure 3A). The majority of these purine binders had RNA/DNA binding function (34%) and 14% had ATP/GTP binding functions. The protein kinase binding function was reported as 9% and a further 8% had ATPase/GTPase activity. The remaining 4% of the identified purine binders had NAD/FAD binding function. Similarly, in the N1 group, 64% of the Bis-bound proteins were reported to have purine binding functions, of which 20% had ATP/GTP and RNA/DNA binding functions (Figure 3B). The Protein kinase and NAD/FAD binding functions were similar to the control group, whereas the ATPase/GTPase activity was increased to 12% compared to the control group (S0). In the N6 group, 68% of the Bis-bound proteins had purine binding functions with 21% annotated as having ATP/GTP binding function and 22% annotated as having RNA/DNA binding function (Figure 3C). The protein kinase binding and ATPase/GTPase activity of the purine binders was 12% and 10%, respectively, with NAD/FAD binding functions (3%) similar to the control group. Finally, in the N12 group 63% of the Bis-bound proteins were reported to have purine binding functions, the majority of which are annotated to have ATP/GTP binding function (29%) (Figure 3D). In total, 19% of the purine binders are annotated as having RNA/DNA binding and 11% are annotated as having protein kinase binding functions. NAD/FAD binding functions of the purine binders were similar to the control group; however, no proteins listed as having ATPase/GTPase activity were reported in this group.

### 2.3. Enzymes Captured by the Bis-Probe

We also evaluated the captured proteins at each time point of BME treatment to identify enzymes. The Bis-probe captured 42 distinct enzymes (Supplementary Table S1) that were identified in the entire data set (S0, N1, N6 and N12). In total, 20 enzymes were identified in the control group, 23 in the N1 group, 22 in the N6 group and 24 enzymes were identified in the N12 groups (Figure 3). Among the distinct enzymes captured by the Bis-probe, E3 ubiquitin-protein ligase and ADP/ATP translocase 1 were probably unique to the control group, S0. Similarly, ATP-dependent RNA helicase DDX1, Transitional endoplasmic reticulum ATPase and probable helicase with zinc finger domain enzymes were uniquely identified in the N1 group. The N6 and the N12 groups had 7 and 6 unique enzymes, respectively, that were captured by the Bis-probe. The unique enzymes identified in the group N6 were fructose-bisphosphate aldolase A, tripeptidyl-peptidase 2, interleukin enhancer-binding factor 3, 6-phosphofructokinase type C, ATP-dependent RNA helicase DDX3X, polymerase I and transcript release factor and probable exonuclease mut-7 homolog enzyme. In the group N12, protein kinase C theta, Lysozyme C, X-ray repair cross-complementing protein 6, Xaa-Pro aminopeptidase 1, putative ATP-dependent RNA helicase DHX57 and glycyl-tRNA synthetase were uniquely captured by the Bis-probe. Apart from the unique enzymes, the Bis-probe captured seven enzymes that were common to all the treatment groups, nine enzymes that were present in at least three of the four groups and eight enzymes that were found in any two groups (Figure 3E). The relative abundance of the Bis-captured enzymes in S0, N1, N6 and N12 was determined by calculating the NSAF values for each of the identified proteins.

### 2.4. Identified Enzymes

**Figure 4:**
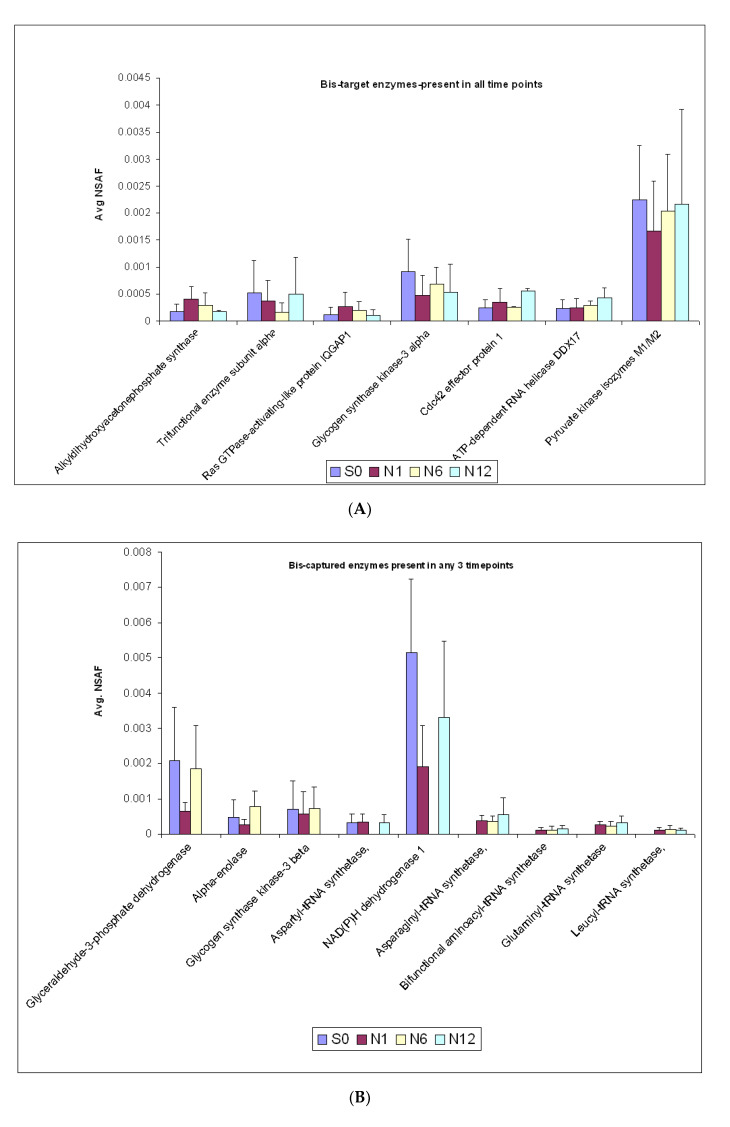

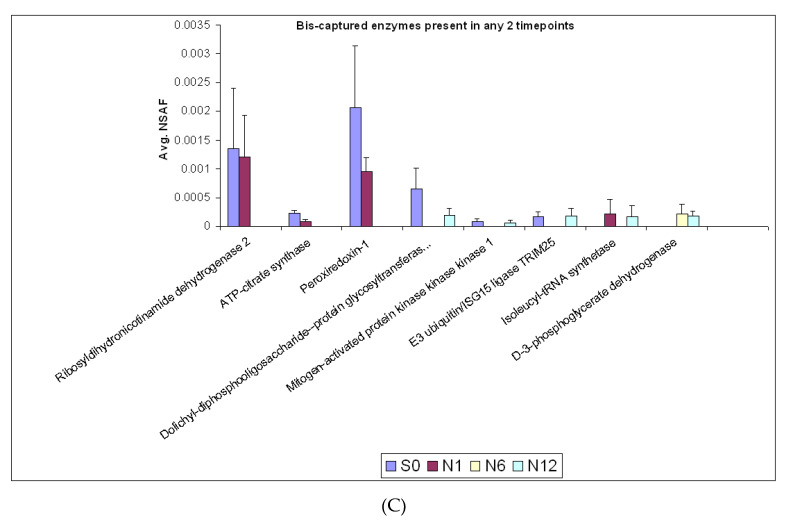
(**A**) Enzymes captured by the Bis-probe in all groups. Statistical analysis of the abundance levels of the proteins present in all the groups was analysed by a one-way ANOVA analysis. (**B**) Bis-bound enzymes present in any three time points Statistical analysis of the abundance levels of the proteins present in all the groups was analysed by a one-way ANOVA analysis. (**C**) Bis-bound enzymes present in any two time points. Statistical analysis of the abundance levels of the proteins present in all the groups was performed by a one-way ANOVA analysis.

In this study we identified seven enzymes that were captured by the Bis-probe in all the different groups (Figure 4A). These included the neurite growth promoting enzymes Ras GTPase-activating-like protein IQGAP1, glycogen synthase kinase-3 alpha and cdc42 effector protein 1. The Bis-probe also captured the metabolic enzymes Pyruvate kinase isozymes M1/M2, alkyldihydroxyacetonephosphate synthase and trifunctional enzyme subunit alpha in all the groups. No statistically significant changes were observed in the relative abundance of these Bis-bound enzymes across the various groups using ANOVA analysis of the log NSAF values.

We also identified nine enzymes that were captured by the Bis-probe in three different time points of BME treatment and absent in one. Of these nine enzymes, glyceroaldehyde 3-phosphate dehydrogenase (GAPDH), alpha enolase and glycogen synthase kinase-3 beta were identified only in the control (S0), N1 and N6 groups, but were absent in the N12 group. Further, the relative abundance of the proteins was similar in the S0 and the N6 groups. However, the N1 group had the lowest abundance of GAPDH (Figure 4B). Similarly, Aspartyl-tRNA synthetase and NAD(P)H dehydrogenase were only captured in the S0, N1 and N12 groups and were absent in the N6 groups. Interestingly, most of the enzymes involved in protein biosynthesis, asparaginyl-tRNA synthetase, bifunctional aminoacyl-tRNA synthetase, glutaminyl-tRNA synthetase, leucyl-tRNA synthetase, that were captured by the Bis-probe were present only in the N1, N6, N12 groups and were absent in the control group.

Similarly, the Bis-probe captured 8 enzymes that were present in only two time points and were absent in the others (Figure 4C). In the control and N1 groups, the Bis-probe captured ribosyldihydronicotinamide dehydrogenase 2, ATP-citrate synthase and Peroxiredoxin-1, which were absent in the N6 and N12 groups. Similarly, dolichyl-diphosphooligosaccharide–protein glycosyltransferase, Mitogen-activated protein kinase 1 and E3 ubiquitin/ISG15 ligase TRIM25 were present only in the control and the N12 groups. Interestingly, Isoleucyl-tRNA synthetase was found only in the N1 and N12 groups and D-3-phosphoglycerate dehydrogenase was only in the N6 and N12 groups.

### 2.5. Enzymes Involved in Neurogenic Differentiation

**Figure 5:**
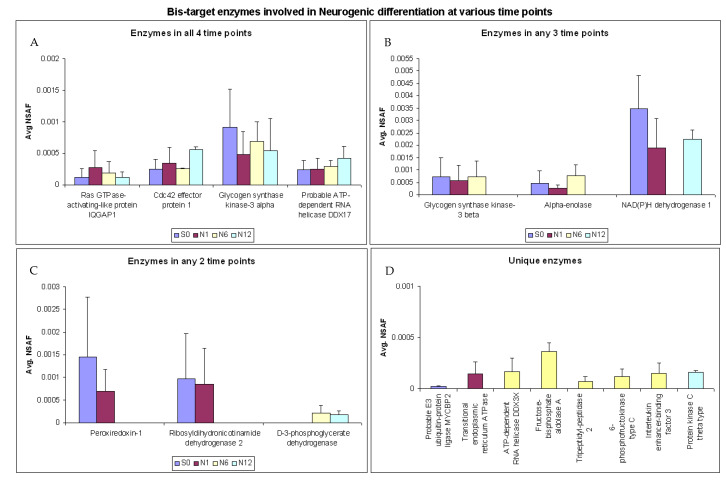
Bis-target enzymes involved in neurogenic differentiation. (**A**) Enzymes present in 4 time points, (**B**) enzymes in any 3 time points, (**C**) enzymes present in any 2 time points (**D**) unique enzymes.

In this study, we identified 18 enzymes that were captured by the Bis-probe and were involved in neurogenic differentiation. Of these, Ras GTPase-activating-like protein IQGAP1, cdc42 effector protein 1, glycogen synthase kinase-3 alpha and probable ATP-dependent RNA helicase DDX17 were identified in all the time points without any significant difference in their relative abundance (Figure 5A). Glycogen synthase kinase-3 beta and alpha-enolase were found consistently in S0, N1 and N6 groups but were absent in the N12 group, whereas NAD(P)H dehydrogenase 1 was present only in the S0, N1 and N12 but was absent in the N6 group (Figure 5B). Further, Peroxiredoxin-1 and ribosyldihydronicotinamide dehydrogenase 2 could be identified at only two time points (S0 and N1). In contrast, D-3-phosphoglycerate dehydrogenase was present only in N6 and N12 groups but absent in S0 and N1 groups (Figure 5C). The Bis-probe also captured enzymes that were uniquely observed only at a particular time point and were absent in the rest of the treatments. These included Probable E3 ubiquitin-protein ligase MYCBP2, Transitional endoplasmic reticulum ATPase and Protein kinase C theta type, that were found only in groups S0, N1 and N12, respectively. The N6 group had majority of the enzymes involved in neurogenic differentiation that were unique to this group. Five enzymes (Figure 5D, ATP-dependent RNA helicase DDX3X, fructose-bisphosphate aldolase A, tripeptidyl-peptidase 2, 6-phosphofructokinase type C and Interleukin enhancer-binding factor 3, were captured by the Bis-probe that were unique to the N6 group and were involved in neurogenic differentiation.

### 2.6. Protein–Protein Interaction Network Analysis of Bis-Enriched hADSC and Neural Differentiated Proteomes

The confirmed Bis-probe captured proteins were evaluated in the interaction network software Cytoscape (v3.8.0) [22] to evaluate their interactive role in the differentiation process. Data input was searched against the well-maintained open access databases, Gene Ontology, SwissProt, Ensembl, TrEMBL, UniProt, PDB, EBI, ENTREZ and RCSB. The visualized result was constructed as an interactive graphical layout. Proteins within the interaction network are represented as nodes and the interactions linking the nodes are lines known as edges. The organic layout utilised applies a force-directed algorithm model which treats each node within the network as a physical object with attraction and repulsion forces dependent on node proximity [22,23]. This evenly distributes nodes and limits overlapping edge intersections, allowing unambiguous edge analysis [24]. The organic layout shows the biological clustering structure of a graph. Each group’s uniquely expressed protein nodes at the time points S0, N1, N6 and N12 were designated a specific colour (red, green, yellow and purple, respectively) for an interaction network differential display. The temporal progression of the networks allows the carryover of time specific nodes throughout all networks. Proteins that were not present in later time point data sets were no longer visualized in the networks. Each network was directionally aligned and overlayed for differential network analysis. The two largest subgroups identified as the "ribosomal" and "structural" clusters intercept a significant number of interactions across the network via several hub proteins. The time-point-specific expressed protein nodes have varying effects on the overall layout of each network, dependent on the types of interactions formed.

**Figure 6:**
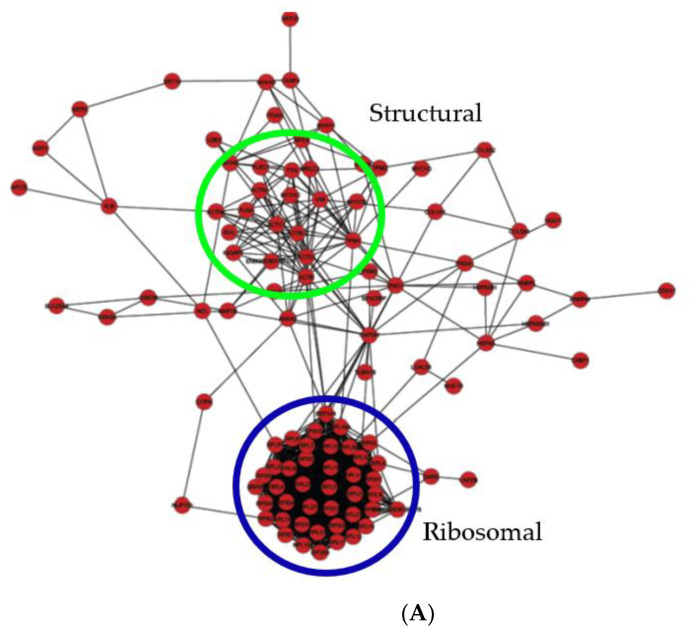

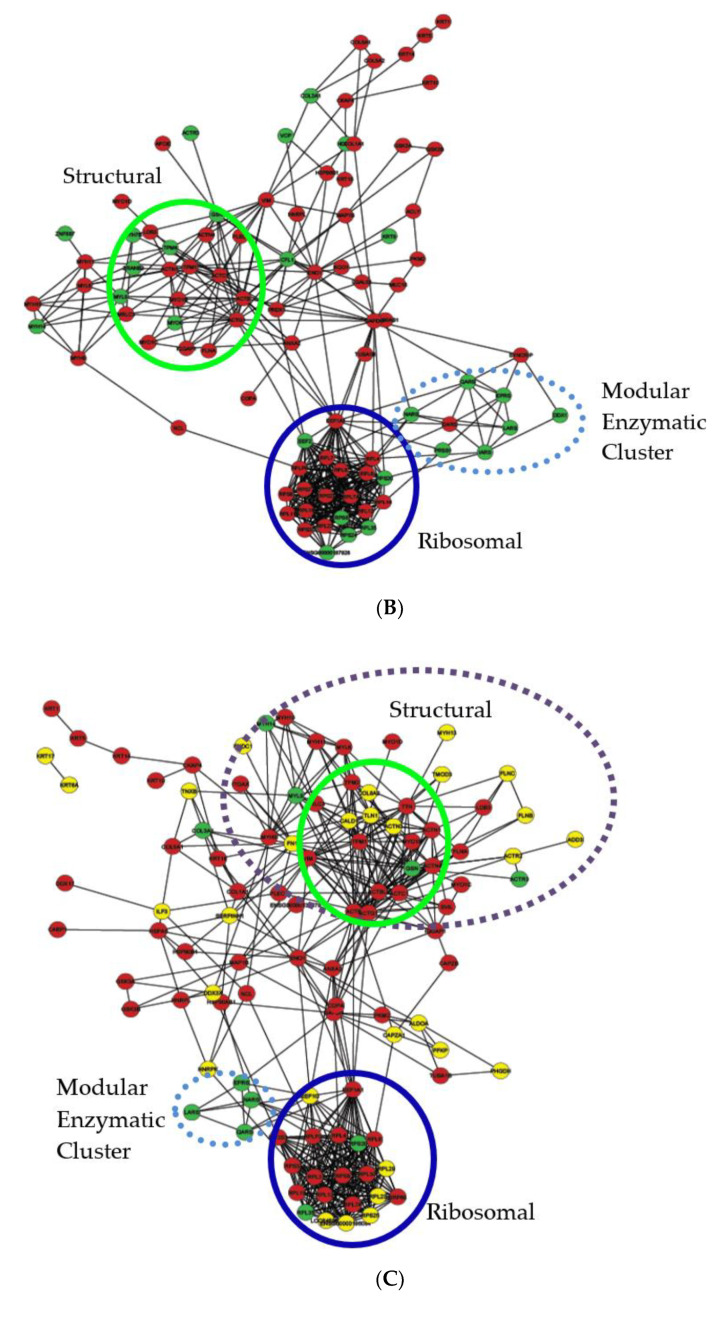

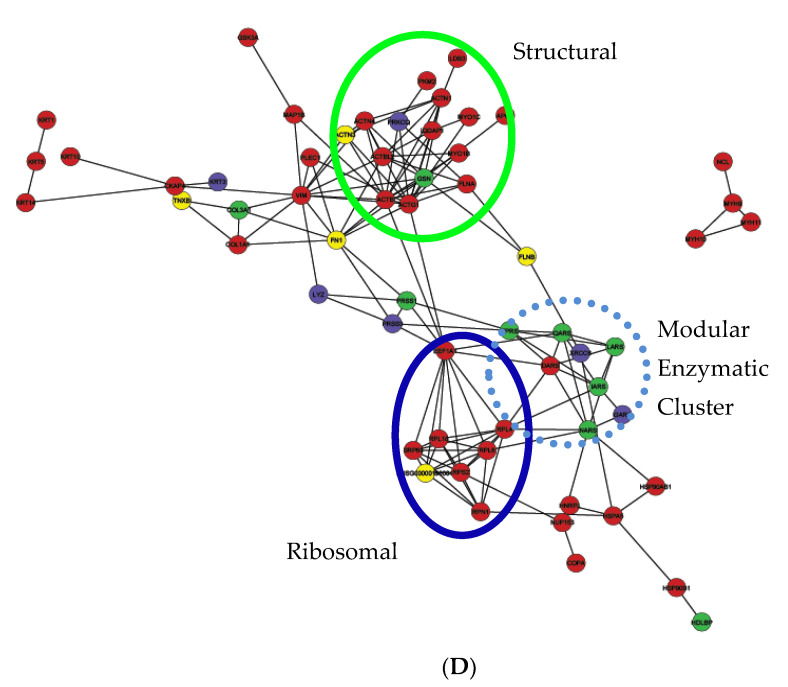
(**A**) Pre-induction media 1h (S0). Proteins are represented by coloured circular nodes. In total, 105 Red nodes denote S0 specific proteins. Connecting lines between each node indicate annotated interactions between identified proteins. Ribosomal cluster is encircled blue and the structural cluster is encircled green. (**B**) hADSCs differentiation media 1h (N1). Proteins are represented by coloured circular nodes. In total, 65 Red nodes denote shared S0 proteins from 5A. In total, 27 Green nodes denote N1 specific proteins. Connecting lines between each node indicate annotated interactions between identified proteins. *Ribosomal cluster* is encircled navy blue, the *structural cluster* is encircled green and *modular enzymatic cluster* is in the light blue dotted ring. (**C**) hADSCs differentiation media 6h (N6) Proteins are represented by coloured circular nodes. In total, 68 Red nodes shared S0 proteins from 5A. In total, 11 Green nodes shared N1 proteins from 5B. In total, 29 Yellow nodes are N6 specific proteins. Connecting lines between each node indicate annotated interactions between identified proteins. Ribosomal cluster is encircled blue, the structural cluster is encircled green and the modular enzymatic cluster is in the light blue dotted ring. (**D**) hADSCs differentiation media 12h (N12). Proteins are represented by coloured circular nodes. Red nodes shared S0 proteins from 5A. Green nodes shared N1 proteins from 5B. In total, 29 Yellow nodes are shared N6 proteins from 5C. Purple nodes are N12 specific proteins. Connecting lines between each node indicate annotated interactions between identified proteins. *Ribosomal cluster* is encircled blue, the *structural cluster* is encircled green and the *modular enzymatic cluster* is in the light blue dotted ring.

Network S0 (Figure 6A) contains 105 protein nodes from the 134 probe captured proteins. The remaining 29 proteins are not visualised as they do not have database confirmed interactions within this dataset. From the 105 nodes, there are 1009 annotated confirmed edge interactions across the network. The “ribosomal” and “structural” clusters, encircled blue and green, respectively, are focal points of the network. The ribosomal cluster contains 42 nodes and 853 adjacent edges. Twenty-eight of the ribosomal proteins are limited to cluster-specific interactions, while the remaining fourteen, 40S ribosomal protein S2, S5, S14, S15a, 60S ribosomal protein PL0, L4, L7, L8, L18a, L19, L30, L32, elongation factor-1 alpha 1 (EEF1A1) and dolichyldiphosphooligosaccharide protein glycosyltransferase (RPN1), interact beyond the ribosomal cluster. These are linked to eleven hub proteins, most notably the enzymes glyceraldehyde-3-phosphate dehydrogenase (GAPDH), alpha-enolase (ENO1) and the structural proteins beta-actin (ACTB), gamma-actin (ACTG1) and tropomyosin-1 alpha chain (TPM1), all of which have in excess of 20 interactions per node. The eleven hub nodes are functional linkers situated between ribosomal to structural clusters also serving numerous functional roles across the network. The structural cluster is much more diffuse, containing 21 nodes and 111 adjacent edges; it is constituted of various actins, tubulins and myosins. There are four identified direct interactions between structural cluster nodes and the ribosomal cluster nodes. GAPDH-, ENO1-, TPM1-, ANAX2- and NCL-associated hub nodes relay as secondary edges between the clusters, interacting with 59 separate nodes network wide.

The overall network logistics of N1 are altered somewhat relative to S0 in that there are 92 nodes with 425 associated edges, of which 194 edges are new. The N1 network shares only 65 proteins with S0, with 40 red nodes no longer being present. There are, however, 27 new N1 specific nodes detected. The introduction of these N1 nodes influences the organic layout due to the fluctuating number of connections. The structural cluster tends directionally toward the left of the network, due to losing 12 S0 nodes and including six new actin and myosin related members, forming 36 new interactions. The ribosomal cluster has significantly decreased in size, missing 26 ribosomal related proteins. However, the introduction of RPL35, EEF2, RPS 6, RPS 16, RPS 20, RPS 24 proteins forms 105 new interactions with direct links to the pathway altering enzyme hubs GAPDH, IARS, PRDX1 and the structural hub ACTG1. An N1-specific eight-node enzymatic modular cluster (Figure 6B–D) has formed contiguously to the ribosomal cluster. Encircled by the blue dotted ring, the modular cluster comprises six synthases NARS, DARS, QARS, IARS, LARS, EPRS, a trypsin-1 precursor and an ATP-dependent RNA helicase with the majority of the 31 edges linked directly to the ribosomal cluster.

The variation of Bis-captured proteins again affects the network wide layout. The N6 network (Figure 6C) contains 103 nodes, a minuscule change from the 105-node S0 network. However, N6, confirmed at 488 edges, has less than half the interactions of S0. The carryover of only 63-S0 and 11-N1 shared proteins, including 29 new yellow N6-specific nodes, reveals an altered organic layout. The structural cluster is significantly different to S0, comprised of a total of 17 nodes with 170 edges. The cluster is derived from 12 S0 nodes, 1 N1 node and 4 N6 nodes, which form 40 new interactions. The newly introduced peripheral nodes, ACTR2, ACTR3, ACTN3, CALD1, COL8A2, FN1, FNDC1, FLNB, FLNC, ADD3, MYL6, MYH10, MYH13, MYO1D, TLN1, and TMOD3, now visualised within the purple dashed ring, extend structural cluster changes. The greater proportion of newly formed interactions of structure altering and binding related peripheral nodes, reveal a greater trend in cytoskeleton modification in the cells. Further dramatic decrease in the ribosomal cluster has occurred, reducing it to 22 proteins, 14 of the original S0 proteins, 2 from N1 and 6 that are N6 specific. The six new nodes, EEF1G, RPS18, RPS25, RPL23, RPL29 and Ubiquitin, form 101 unique edges. The synthase modular cluster observed in N1 has migrated, flanking the ribosomal cluster on the left due to alternate interactions with adjacent proteins. The enzymes phosphofructokinase type C (PFKP), ATP-dependent RNA helicase (DDX3Y), D-3-phosphoglycerate dehydrogenase (PHGDH) and Fructose-bisphosphate aldolase A (ALDOA) are linked both directly and indirectly to either cluster in tandem.

Network N12 (Figure 6D) shows a 42.8% reduction in visualised nodes to 60 Bis-captured proteins and 85.9% reduction in edges to 142 annotated interactions. The structural cluster is reduced to 15 nodes, of which only one, Protein kinase C theta type, is unique to this time point. Within the N12 network, PKCθ interacts with beta-actin (ACTB), gamma-actin (ACTG), filamin-A (FLNA) and pyruvate kinase isozyme (PKM2). The ribosomal cluster has decreased to only eight members with seven S0 shared nodes and one N6 shared node. EEF1A1 is the sole direct link between the two dominant clusters. Some indirect links are associated via the modular cluster, which at N12 has the equal number of node members as the ribosomal cluster. The modular cluster contains DARS from S0 and EPRS, IARS, LARS, NARS, QARS from N6 with the new additions Glycyl-tRNA synthetase (GARS) and ATP-dependent DNA helicase 2 subunit 1 (XRCC6). GARS shares two edges with two modular nodes, while XRCC6 interacts with Filamin-B (FLNB) and the 78 kDa glucose-regulated protein precursor (HSPA5), revealing indirect links of the structural and ribosomal clusters through the modular cluster. Proximal to the modular cluster are a group of nodes, COPA, HDLBP, HNRPL, HSP90AB1, HSP90B1, HSPA5 and NUP153, which in all previous networks were extensively associated with both major clusters. At N12 this group shares four interactions equally between the ribosomal and minor modular cluster. The dissociated quartet of NCL, MYH9, MYH10 and MYH11 no longer share any links with the rest of the network as their functional partners do not exist at this time point.

## 3. Discussion

This study successfully employed a Bis-probe to capture a broad spectrum of purine binding enzymes or structurally related analogues as cofactor for catalysis in hADSCs treated with the neural induction media containing BME. The attachment of a suitable linker and an appropriate affinity capture reagent to bisindolylmaleimides has facilitated the subsequent mass-spectrometry (MS) characterisation of cellular binding targets in previous studies using cell culture-based models in PC12 cells [25], HeLa cells [26,27,28], and in a basal breast cancer cell model [21]. We are the first to report the MS-based characterisation of the binding targets of bisindolylmaleimide in BME-treated hADSCs, adopting our previously described chemical proteomics method, which is compatible with batch-mode analysis of protein obtained from small-scale culture. Studies were conducted on hADSCs treated with BME for 1, 6 and 12 h durations, utilising 100 µg of protein which enabled rapid investigation of stem cell proteomics using Bis-probes. Furthermore, the successful integration of label free quantitation into our small scale quantitative chemical proteomics enabled us to quantitate the relative abundance of the Bis-captured proteins at different time points during BME treatment of hADSCs. This approach allowed for a rapid quantitative evaluation of chemical probes in stem cell models, where protein yields after chemical treatment for neurogenic differentiation are considerably low, unlike aggressively growing cancer cell models. Due to the large number of proteins identified in this study, it is difficult to explore each in detail; the discussion will therefore focus on the enzyme class of proteins, focusing on some key enzymes identified as hubs in the interaction networks exploring their interactions and pathways formed with other identified proteins.

One difficulty with the use of the Bis-probe is its known promiscuous nature for kinase target selectivity [21,27,29,30]. In our own investigation, we also observed that the Bis-probe interacted with other non-kinase, purine binding proteins with related structural binding pockets in hADSCs. However, despite this, almost 70% of the predicted purine binding proteome was enriched by the Bis-probe at all the different time points of BME treatment of hADSCs. We were also able to identify the majority of the kinase and non-kinase targets that were reported [26,27] and previously confirmed [21] as Bis-binding proteins. Additionally, we were also able to identify the Bis-target kinases, GSK α and GSK β, in the hADSCs.

The alpha isoform of GSK was identified in all the different groups of BME treatment, whereas the beta isoform was identified only in the S0, N1 and N6 groups at relatively similar levels and was absent in the N12 groups. The presence of these different isoforms, as well as their specific roles, was reported previously by Cho et al. when differentiating murine bone marrow derived mesenchymal stem cells [31]. In their study, Cho et al. reported the over expression of GSK β to be potentially responsible for cardiomyocyte differentiation of mesenchymal stem cells and the over expression of GSK α to induce the expression of markers of neuronal differentiation. This observation could offer a possible explanation for the capture of the α isoform in all of the time points taken during the neuronal differentiation of hADSCs with BME. Figure 6A indicates that GSKα and GSKβ are functionally linked to ADP/ATP translocase 1, microtubule-associated protein 1B (MAP1B) and ATP-citrate synthase. GSKα/β acquires ATP from the translocase at the mitochondrial membrane. The subsequent phosphorylation of MAP1B by GSKα/β initiates microtubule assembly and cytoskeletal changes that indicate neurite extension, which is an essential step in neurogenesis [32]. This assertion is further supported by the gene knockout studies performed by Lie et al., which support MAP1B’s role in the development and function of the nervous system [33]. In addition to the above, the phosphorylation of citrate synthase by GSKβ to ATP-citrate synthase is found to be abundant in developing nervous tissue and is involved in the biosynthesis of the neurotransmitter acetylcholine [34]. Although varying levels of GSKα were captured in different groups on the basis of average NSAF values, the relative abundance of the protein at different time points was not statistically significant.

GSKβ is associated with Wnt stimulation and has been found to promote self-renewal and the regeneration of nervous system cells; therefore, it has been thought to be implicated in the neuro-differentiation of hADSCs [33]. Furthermore, in neuronal stem cells derived from the rat subventricular zone, Maurer et al. reported that GSK β is a central regulator for the differentiation and cell survival of adult neuronal stem cells [35]. Maurer et al. also used proteomic approaches, to study the inhibitory effect of GSK β on neuronal differentiation and concluded that GSKβ inhibition results in the participation of Wnt/β- catenin signalling pathways for enhanced neuronal differentiation and activates the transcription of β-catenin target genes. In addition to this, they also reported decreased apoptosis of neural stem cells due to GSK β inhibition. Based on this previous work, in the present study, the lower relative abundance of GSK β observed at S0, N1 and N6 and its absence in the N12 group could be a possible neurogenic and cell survival mechanism.

Additionally, we identified the PKC isoenzyme (PKCθ) uniquely expressed in all three biological replicates of the N12 group. PKCθ has been previously identified to be enriched by Bis-probes in our study of basal breast cancer cells [21]. However, at the time of writing, there are no studies that reported the role of PKCθ in BME-treated hADSCs. Despite this, Sparatore, B. et al. reported evidence for changes in PKCθ distribution and molecular properties during the neuronal differentiation of the rat pheochromocytoma PC12 cell line [36]. They also reported PKCθ involvement in the long-term program of neuronal differentiation and that PKCθ is also associated with the reorganisation of cytoskeletal networks during the differentiation of PC12 cells towards neuron-like non-proliferating phenotypes. In Figure 6D, PKCθ has annotated interactions with pyruvate kinase isozymes M2 (PKM2), beta-actin, gamma-actin and filamin-A (FLNA). Although PKM2 is a muscle isoform, it is widely expressed in some differentiated tissues, including brain tissue and nervous cells [37] though its role in these tissues remains to be elucidated.

In contrast, the hydrolysation of the non-muscle type β and γ type actin allows their polymerisation, forming microfilaments in various tissues, which together with the axonal transport of β-actin mRNA in both primary and transformed neuronal cell lines, has been identified in the development of neuronal growth cones [38]. More specifically, γ-Actin plays a role in the chemotaxis migration of the axon growth cone. Studies have revealed that defects in γ-actin yield damaged neural receptors in the inner ear and nerve pathways to the brain [39], suggesting a greater involvement of enzyme-linked interactions with structural proteins in the differentiation process.

The interaction between PKCθ and FLNA is also implicated in neuroblast migration and neuronal orientation [40], where FLNA is known to promote branching and anchoring of actin filaments in cytoskeleton remodelling. Another study in neuroblastomas and Jurkat cells have reported the role of PKC θ in apoptotic induction [41] showing that apoptosis rates are known to have increased over induction time during chemical induction, using BME in adult rat bone marrow MSCs [39]. Thus, the identification of PKCθ only in the N12 group of the present study could be related to the onset of apoptosis in the 12 h BME-treated hADSCs.

Our study identified MAP3 kinase as a Bis-binding target in hADSCs, where it was identified in the S0 and N12 groups: MAP3Ks are activated by extracellular stimuli which are initiated through cell receptors where signals are typically transduced through small GTPases. Our study has identified Ras GTPase activating-like protein IQGAP1 in all the time points of neuronal differentiation of hADSCs, with the highest expression doubling the baseline after BME treatment, indicating the potential activation of the MAP3K signalling pathway.

IQGAP was previously reported as a Bis-target in HeLa cells [26]. In eukaryotic cells, IQGAP1 binds to and alters the function of several proteins, including actin, E-cadherin, β-catenin, CDC42 and RAC1. Studies have reported IQGAP1 as a major regulatory protein that interacts with the actin cytoskeleton [42] and the effect is mediated both by a direct interaction with actin and indirectly via Cdc42 and Rac1 [43]. Although no reports are available on its detection and cytoskeletal regulation in BME treated hADSCs, Li et al. were the first to report the presence of IQGAP1 in neuronal cells [44]. Their study demonstrated the expression of IQGAP1 in hippocampal neurons from E17 rat embryos and revealed the ubiquitous presence of this enzyme throughout the cells, along the neurites and the developing axon, as well as the growth cone. In addition to this, they confirmed the ability and over expression of this enzyme to promote neurite outgrowth by regulating the neuronal cytoskeleton. Neurite outgrowth requires the interplay of all the three major types of protein filaments that form the cytoskeleton; actin filaments, microtubules and intermediate filaments and the precise regulation of cytoskeletal dynamics is important for neurite outgrowth through the reorganization of cytoskeleton components [45,46]. The overexpression of IQGAP1 has also been demonstrated to increase the amount of endogenous active Cdc42 in mammalian cells, resulting in morphological changes with the production of filopodia and microspikes [43]. Studies have also suggested the role of IQGAP1 as a molecular link between active Cdc42 and the actin cytoskeleton [47,48] which we have confirmed by finding IQGAP1 and Cdc42 in all the time points on the neuronal differentiation of hADSCs.

Apart from the kinases and the GTPases, the Bis-probe also captured the previously reported [21,26,27] non-kinase target of bisindolylmaleimide ribosyldihydronicotinamide dehydrogenase (NQO2) in the S0 and N1 groups. Furthermore, our study also identified NAD(P)H dehydrogenase 1 (NQO1) in the S0, N1 and N12 groups, whereby the relative abundance of the NQO1 dehydrogenase was higher than that of the NQO2 dehydrogenase; neurogenic differentiation agents are known to induce oxidative stress, which activates transcription factors such as Nrf2 and its target gene NQO1 to counteract redox imbalance and eliminate the harmful reactive species [49]. The differentiating agent BME has also been reported to cause oxidative stress in bone marrow stem cells [50], which explains the identification of NQO1 in BME treated hADSCs. However, whether NQO1 has any role in neuronal differentiation has not been investigated. Zhao et al., have reported that the over expression of Nrf2 promoted neuronal differentiation in the neuroblastoma cell line, SH-SY5Y.

The Bis-probe also captured another dehydrogenase, such as D-3 phosphoglycerate dehydrogenase (3PGDH), that was observed only in the N6 and N12 groups. The 3PGDH is an initial step enzyme for de novo L-serine biosynthesis in animal cells and is synthesised from the glycolytic intermediate 3-phosphoglycerate. It is an indispensable precursor for the synthesis of proteins, membrane lipids, nucleotides and neuroactive amino acids, D-serine and glycine [51]. Yamasaki et al. [51] investigated the cellular expression of 3PGDH and also reported the presence of over expression of 3PGDH in neuroepithelial stem cells. Furthermore, emerging evidence indicates that the non-essential amino acid, L-serine, plays an essential role in neuronal development and function. The capture of this enzyme by the Bis-probe at the N6 and N12 time points of the neurogenic differentiation of hADSCs may indicate an active synthesis of this neuronal-development amino acid.

Finally, we also identified the antioxidant enzyme peroxiredoxin 1 (Prdx-1) in the S0 and N1 groups, which were previously reported as Bis-targets in HeLa cells [26]. Prdx-1 are widely expressed hydrogen peroxide scavenger proteins, best known for their role in detoxifying reactive oxygen species, protecting against oxidative stress, DNA damage, and cancer, and have also been suggested to act in cellular signalling and as molecular chaperons [52,53]. This Bis-target has also been recently reported to be involved in a thiol redox signalling cascade that is known to induce neuronal differentiation [54]. Yan, Y. et al. were the first to identify a new role of Prdx-1 in regulating neuronal differentiation, whereby the cascade is mediated by the transmembrane protein glycerophosphodiester phosphodiesterase (GDE2) and the antioxidant protein Prdx1 that controls the timing of motor neuron differentiation in the spinal cord. They utilised an innovative proteomic screening approach to demonstrate the direct binding of Prdx-1 to GDE2, which serves as an activating cofactor that promotes neuronal differentiation.

## 4. Methods

### 4.1. Cell Culture

STEMPRO Human adipose-derived stem cells (hADSCs) were purchased from Life Technologies (Carlsbad, CA) and were cultured in DMEM/F12 glutamax (Life Technologies, Carlsbad, CA, USA), supplemented with 10% FBS (Gibco, Life Technologies, Carlsbad, CA, USA) and 1% antibiotic (penicillin, 100 μg/mL and streptomycin, 100 μg/mL(Gibco, Life Technologies, Carlsbad, CA, USA)) at 37 °C in the presence of 5% CO_2_. For each passage, the cells were plated at about 5000 cells/cm^2^ and grown to confluence in a T175 Nunc flask.

### 4.2. Neurogenic Differentiation

Sub-confluent hADSCs at passage 3 were cultured for up to 12 h in 15 mL pre-induction medium, made up of DMEM/10% FBS/1 mM β-mercaptoethanol (BME). Neuronal differentiation was then induced for up to 12 h in a neuronal induction media (DMEM/10 mM BME) [5,8].

### 4.3. Sample Preparation

Three independent populations of pre-induced cells (S0) were harvested along with neuronal induced cells at three different time points 1 h (N1), 6 h (N6) and 12 h (N12). After rinsing twice in 10 mL 1× sterile PBS, the cells were lysed in Pierce IP lysis buffer (Life Technologies Thermofischer, Carlsbad, CA, USA)), containing 25 mM Tris-HCl, pH 7.4, 150 mM NaCl, 1% NP-40, 1 mM EDTA and 5% glycerol, plus protease inhibitors (1 mM phenylmethylsulfonyl fluoride, 1 mM EDTA, 1 µg/mL aprotinin, 1 µg/mL leupeptin, 1 µg/mL pepstatin) and phosphatase inhibitors (1 mM sodium fluoride and 1 mM sodium orthovanadate). Cell lysates were vortexed and clarified by centrifugation at 14,000 rpm for 5 min at 4 °C. The clarified lysates were filtered through Zeba Spin Desalting Columns (Life Technologies Thermofischer, Carlsbad, CA, USA) into fresh lysis buffer. The cell lysates were desalted to remove endogenous nucleotides, particularly ATP, which competes with the probe for the ATP binding sites of target proteins. Relative protein concentration was determined using a bicinchoninic acid (BCA) assay.

### 4.4. Synthesis of Bis-probe, Affinity Pull down, In-gel Clean up and Digestion

The Bis-Probe consisted of a Bisindolylmaleimide reactive group for affinity binding and a biotin reporter group with a spatial polyethelene oxide (PEO) linker, and was synthesised as described previously [21]. For affinity pull down experiments, 100 µg protein from each independent population of pre-induced cells (S0) and neuronal induced cells at three different time points, 1 h (N1), 6 h (N6) and 12 h (N12), was volume adjusted to 500 µL with Pierce IP lysis buffer. The remaining affinity pull down, in-gel clean up and digestion steps were completed as previously described in [17].

### 4.5. Mass Spectrometry, Protein Identification and Data Analysis

The tryptic peptide samples obtained for each of the independent biological replicates at time points S0, N1, N6 and N12 were analysed by nanoLC-MS/MS using an LTQ XL linear ion-trap mass spectrometer (Life Technologies Thermofischer, Carlsbad, CA, USA), as described previously [21]. Data files were processed and searched against the SwissProt Human database using the Global Proteome Machine (GPM) (version 2.1.1) and the X!Tandem algorithm [55]. The search modifications and parameters used were the same as described previously [21]. The proteins identified for all the replicates for each of the four time points were further filtered to remove proteins found in fewer than two biological replicates (true identifications). After this filtering, the peptide and protein false discovery rates (FDR) were calculated. Peptide FDR was calculated as 2 × (total number of peptides representing reversed protein hits in the list/total number of peptides representing all proteins in the list) × 100, and protein FDR was calculated as (number of reverse protein hits in a list/ total number of proteins in the list) × 100.

### 4.6. Calculation of Protein Normalized Spectral Abundance Factor (NSAF)

Normalized spectral abundance factors (NSAFs) were calculated for each protein in all the groups using the formula: NSAF = (Spc/L)/∑(Spc/L), described by Zybailov et al. [56], where Spc refers to the spectral count (total number of MS/MS spectra) identifying a given protein, L is the length of the protein and ∑(Spc/L) refers to the summation of spectral count/length of all the proteins identified in the experiment. Protein identifications were only included in NSAF data analysis if a given protein was identified in at least 2 of the 3 biological replicates. Further, a spectral fraction of 0.5 was added to all values to compensate for null values [56]. Statistical analysis of the abundance levels of the proteins present in all the groups was analysed by a one-way ANOVA analysis of the log NSAF data. To examine the natural log NSAF data, overlapped kernel plots were generated and this assured the normal distribution of data for each group.

## 5. Conclusions

This study successfully applied and used a bisindolylmaleimide chemical probe to enrich and capture specific classes of proteins in the neural differentiation of hADSCs for quantification. The roles of classes of proteins involved in enzymatic reactions, structural rearrangement and ribosomal interactions have been identified and their roles during the temporal differentiation time points explored. The results support the notion that a small reductive molecule, such as BME, in short, controlled, and temporal treatment intervals of MSCs, has the potential to induce the expression of enzymes that initiate early mechanisms and pathways for pre-neural differentiation.

## Figures and Tables

**Table 1:** The number of Bis-probe captured proteins as well as protein and peptides FDR identified after liquid chromatography-tandem mass spectrometry analysis ADSCs and ADSCs treated with BME in each respective timepoint.

Groups	Number of Proteins	Protein FDR	Peptide FDR
S0	133	0.11949686	0.0082224
N1	118	0.17567568	0.0106044
N6	127	0.15584416	0.0076948
N12	89	0.11949686	0.0082224

## Data Availability

The data presented in this study are available on request from the corresponding author.

## Supplementary Materials

The following are available online at https://www.mdpi.com/1422-0067/22/1/160/s1.

## Abbreviations

BME	Beta-mercaptoethanol
DMEM	Delbuccos modified eagle medium
FBS	Fetal bovine serum
FDR	False discovery rate
hADSCs	Human adipose-derived stem cells
LTQ XL	Linear Ion trap mass spectrometer
MSC	Mesenchymal stem cells
NSAF	Normalized spectral abundance factors
PEO	Polyethelene oxide
PIM	Pre-induction media
SVF	Stromal vascular fraction
