# Supplementary material for: Quantitative Proteomic Profiling of Small Molecule Treated Mesenchymal Stem Cells Using Chemical Probes
Source: Int J Mol Sci. 2020 Dec 26;22(1):160. doi: 10.3390/ijms22010160 (PMC7795898; doi:10.3390/ijms22010160)
Supplement: Supplementary file 1 [file ijms-22-00160-s001.pdf]

Supplementary Table 1 The identified Bis-probe captured enzymes after liquid chromatography-tandem mass spectrometry analysis of ADSCs treated with BME.

Protein names	Accession Number	Gene names	Mass (Da)	Length	Gene ontology (biological process)	Gene ontology (molecular function)
**Dolichyl-diphosphooligosaccharide--protein glycosyltransferase subunit 1**	P04843	RPN1	68,569	607	cellular protein modification process [GO:0006464]<br>protein N-linked glycosylation [GO:0006487]<br>protein N-linked glycosylation via asparagine [GO:0018279]	RNA binding [GO:0003723]
**Ras-related protein Rab-21**	Q9UL25	RAB21 KIAA0118	24,348	225	anterograde axonal transport [GO:0008089]<br>regulation of axon extension [GO:0030516]	GDP binding [GO:0019003]; GTPase activity [GO:0003924]; GTP binding [GO:0005525]
**E3 ubiquitin/ISG15 ligase TRIM25**	Q14258	TRIM25 EFP RNF147 ZNF147	70,973	630	interferon-gamma-mediated signaling pathway [GO:0060333]	cadherin binding [GO:0045296]; ligase activity [GO:0016874]; metal ion binding [GO:0046872]; RIG-I binding [GO:0039552]; RNA binding [GO:0003723]; ubiquitin protein ligase activity [GO:0061630]
**D-3-phosphoglycerate dehydrogenase**	O43175	PHGDH PGDH3	56,651	533	brain development [GO:0007420]<br>glial cell development [GO:0021782]<br>neural tube development [GO:0021915]<br>neuron projection development [GO:0031175]	electron transfer activity [GO:0009055]; L-malate dehydrogenase activity [GO:0030060]; NAD binding [GO:0051287]; phosphoglycerate dehydrogenase activity [GO:0004617]
**Cdc42 effector protein 1**	Q00587	CDC42EP1 BORG5 CEP1 MSE55	40,295	391	positive regulation of actin filament polymerization [GO:0030838]	cadherin binding involved in cell-cell adhesion [GO:0098641]; GTP-Rho binding [GO:0017049]
**Mitogen-activated protein kinase kinase kinase 1**	Q13233	MAP3K1 MAPKKK1 MEKK	164,470	1512	protein phosphorylation [GO:0006468]	ATP binding [GO:0005524]; MAP kinase kinase kinase activity [GO:0004709]; protein kinase activity [GO:0004672];

		MEKK1				protein kinase binding [GO:0019901];
**ADP/ATP translocase 1**	P12235	SLC25A4 ANT1	33,064	298	ADP transport [GO:0015866]	adenine transmembrane transporter activity [GO:0015207]; ATP transmembrane transporter activity [GO:0005347]
**NAD**	P15559	NQO1 DIA4 NMOR1	30,868	274	positive regulation of neuron apoptotic process [GO:0043525]	cytochrome-b5 reductase activity, acting on NAD(P)H [GO:0004128]; identical protein binding [GO:0042802]; NAD(P)H dehydrogenase (quinone) activity [GO:0003955]; oxidoreductase activity [GO:0016491]; RNA binding [GO:0003723]; superoxide dismutase activity [GO:0004784]
**Peroxiredoxin-1**	Q06830	PRDX1 PAGA PAGB TDPX2	22,110	199	cell population proliferation [GO:0008283]	cadherin binding [GO:0045296]; identical protein binding [GO:0042802]; peroxidase activity [GO:0004601]; RNA binding [GO:0003723]; thioredoxin peroxidase activity [GO:0008379]
**Protein kinase C theta type**	Q04759	PRKCQ PRKCT	81,865	706	axon guidance [GO:0007411] regulation of megakaryocyte differentiation [GO:0045652]	ATP binding [GO:0005524]; calcium-dependent protein kinase C activity [GO:0004698]; metal ion binding [GO:0046872]; protein kinase activity [GO:0004672]; protein serine/threonine kinase activity [GO:0004674]
**Glycogen synthase kinase-3 beta**	P49841	GSK3B	46,744	420	negative regulation of calcineurin-NFAT signaling cascade [GO:0070885] negative regulation of dopaminergic neuron differentiation [GO:1904339] negative regulation of neuron death [GO:1901215] neuron projection development [GO:0031175]	ATP binding [GO:0005524]; beta-catenin binding [GO:0008013]; dynactin binding [GO:0034452]; kinase activity [GO:0016301]; NF-kappaB binding [GO:0051059]; p53 binding [GO:0002039]; protease binding [GO:0002020]; protein kinase A catalytic subunit binding [GO:0034236]; protein kinase activity [GO:0004672]; protein kinase binding [GO:0019901]; protein serine/threonine kinase activity

					neuron projection organization [GO:0106027] positive regulation of neuron death [GO:1901216] regulation of axon extension [GO:0030516] regulation of axonogenesis [GO:0050770] regulation of neuron projection development [GO:0010975] Wnt signaling pathway [GO:0016055]	[GO:0004674]; RNA polymerase II transcription factor binding [GO:0001085]; tau protein binding [GO:0048156]; tau-protein kinase activity [GO:0050321]; ubiquitin protein ligase binding [GO:0031625]
**Glycogen synthase kinase-3 alpha**	P49840	GSK3A	50,981	483	positive regulation of neuron apoptotic process [GO:0043525] regulation of neuron projection development [GO:0010975] Wnt signaling pathway [GO:0016055]	ATP binding [GO:0005524]; protein kinase A catalytic subunit binding [GO:0034236]; protein serine/threonine kinase activity [GO:0004674]; signaling receptor binding [GO:0005102]; tau protein binding [GO:0048156]; tau-protein kinase activity [GO:0050321]
**Ras GTPase-activating-like protein IQGAP1**	P46940	IQGAP1 KIAA0051	189,252	1657	neuron projection extension [GO:1990138]	actin filament binding [GO:0051015]; cadherin binding [GO:0045296]; calcium ion binding [GO:0005509]; calmodulin binding [GO:0005516]; GTPase activator activity [GO:0005096]; GTPase inhibitor activity [GO:0005095]; MAP-kinase scaffold activity [GO:0005078]; mitogen-activated protein kinase binding [GO:0051019
**ATP-citrate synthase**	P53396	ACLY	120,839	1101	acetyl-CoA biosynthetic process [GO:0006085]	ATP binding [GO:0005524]; ATP citrate synthase activity [GO:0003878]; metal ion binding [GO:0046872]
**E3 ubiquitin-protein ligase MYCBP2**	O75592	MYCBP2 KIAA0916 PAM	513,636	4678	branchiomotor neuron axon guidance [GO:0021785]	guanyl-nucleotide exchange factor activity [GO:0005085]; identical protein binding [GO:0042802]; Ran GTPase

					central nervous system projection neuron axonogenesis [GO:0021952] neuromuscular process [GO:0050905] regulation of axon guidance [GO:1902667]	binding [GO:0008536]; ubiquitin protein ligase activity [GO:0061630]; zinc ion binding [GO:0008270]
**Ribosyldihydronicotinamide dehydrogenase [quinone]**	P16083	NQO2 NMOR2	25,919	231	memory [GO:0007613] positive regulation of neuron apoptotic process [GO:0043525]	chloride ion binding [GO:0031404]; dihydronicotinamide riboside quinone reductase activity [GO:0001512];
**Glycine--tRNA ligase**	P41250	GARS1 GARS	83,166	739	diadenosine tetraphosphate biosynthetic process [GO:0015966]	ATP binding [GO:0005524]; bis(5'-nucleosyl)-tetraphosphatase (asymmetrical) activity [GO:0004081]; glycine-tRNA ligase activity [GO:0004820]; identical protein binding [GO:0042802];
**Lysozyme C**	P61626	LYZ LZM	16,537	148	cellular protein metabolic process [GO:0044267]	identical protein binding [GO:0042802]; lysozyme activity [GO:0003796]
**Glyceraldehyde-3-phosphate dehydrogenase**	P04406	GAPDH GAPD CDABP0047 OK/SW-cl.12	36,053	335	neuron apoptotic process [GO:0051402]	aspartic-type endopeptidase inhibitor activity [GO:0019828]; disordered domain specific binding [GO:0097718]; glyceraldehyde-3-phosphate dehydrogenase (NAD+) (phosphorylating) activity [GO:0004365];
**Pyruvate kinase PKM**	P14618	PKM OIP3 PK2 PK3 PKM2	57,937	531	animal organ regeneration [GO:0031100]	ADP binding [GO:0043531]; ATP binding [GO:0005524]; cadherin binding [GO:0045296]; identical protein binding [GO:0042802]; kinase activity [GO:0016301]; magnesium ion binding [GO:0000287];
**Alkyldihydroxyacetonephosphate synthase, peroxisomal**	O00116	AGPS AAG5	72,912	658	ether lipid biosynthetic process [GO:0008611]	alkylglycerone-phosphate synthase activity [GO:0008609]; FAD binding [GO:0071949]; oxidoreductase activity [GO:0016491]
**Putative ATP-dependent RNA**	Q6P158	DHX57	155,604	1386		ATP binding [GO:0005524]; metal ion

**helicase DHX57**						binding [GO:0046872]; RNA binding [GO:0003723]; RNA helicase activity [GO:0003724]
**Alpha-enolase**	P06733	ENO1 ENO1L1 MBPB1 MPB1	47,169	434	positive regulation of ATP biosynthetic process [GO:2001171]	cadherin binding [GO:0045296]; DNA-binding transcription repressor activity, RNA polymerase II-specific [GO:0001227]; GTPase binding [GO:0051020]; magnesium ion binding [GO:0000287]; phosphopyruvate hydratase activity [GO:0004634
**ATP-dependent RNA helicase DDX3X**	O00571	DDX3X DBX DDX3	73,243	662	cell differentiation [GO:0030154] positive regulation of canonical Wnt signaling pathway [GO:0090263] Wnt signaling pathway [GO:0016055]	ATPase activity [GO:0016887]; ATP binding [GO:0005524]; cadherin binding [GO:0045296]; CTPase activity [GO:0043273]; DNA binding [GO:0003677]; DNA helicase activity [GO:0003678];
**Probable ATP-dependent RNA helicase DDX17**	Q92841	DDX17	80,272	729	alternative mRNA splicing, via spliceosome [GO:0000380] androgen receptor signaling pathway [GO:0030521]	ATP binding [GO:0005524]; RNA binding [GO:0003723]; RNA-dependent ATPase activity [GO:0008186]; RNA helicase activity [GO:0003724]; transcription coactivator activity [GO:0003713]
**Fructose-bisphosphate aldolase A**	P04075	ALDOA ALDA	39,420	364	actin filament organization [GO:0007015]	actin binding [GO:0003779]; cadherin binding [GO:0045296]; cytoskeletal protein binding [GO:0008092]
**Exonuclease mut-7 homolog**	Q8N9H8	EXD3 HBE269	96,598	876		3'-5' exonuclease activity [GO:0008408]; metal ion binding [GO:0046872]; nucleic acid binding [GO:0003676]
**Probable helicase with zinc finger domain**	P42694	HELZ DRHC KIAA0054	218,970	1942	post-transcriptional gene silencing by RNA [GO:0035194]	ATP binding [GO:0005524]; helicase activity [GO:0004386]; metal ion binding [GO:0046872]; RNA binding [GO:0003723]
**Bifunctional glutamate/proline--tRNA ligase**	P07814	EPRS1 EPRS GLNS PARS QARS QPRS PIG32	170,591	1512	cellular response to insulin stimulus [GO:0032869]	ATP binding [GO:0005524]; glutamate-tRNA ligase activity [GO:0004818]; GTPase binding [GO:0051020]; identical protein binding [GO:0042802]; proline-

					cellular response to interferon-gamma [GO:0071346] glutamyl-tRNA aminoacylation [GO:0006424]	tRNA ligase activity [GO:0004827];
**Leucine--tRNA ligase, cytoplasmic**	Q9P2J5	LARS1 KIAA1352 LARS	134,466	1176	cellular response to leucine [GO:0071233] glutaminyl-tRNA aminoacylation [GO:0006425] positive regulation of GTPase activity [GO:0043547]	aminoacyl-tRNA editing activity [GO:0002161]; ATP binding [GO:0005524]; glutamine-tRNA ligase activity [GO:0004819]; GTPase activator activity [GO:0005096]; leucine-tRNA ligase activity [GO:0004823]
**Asparagine--tRNA ligase, cytoplasmic**	O43776	NARS1 NARS NRS	62,943	548	cell migration [GO:0016477]	asparagine-tRNA ligase activity [GO:0004816]; ATP binding [GO:0005524]; CCR3 chemokine receptor binding [GO:0031728];
**Aspartate--tRNA ligase, cytoplasmic**	P14868	DARS1 DARS PIG40	57,136	501	aspartyl-tRNA aminoacylation [GO:0006422] protein-containing complex assembly [GO:0065003]	aminoacylase activity [GO:0004046]; aspartate-tRNA ligase activity [GO:0004815]; ATP binding [GO:0005524]; RNA binding [GO:0003723]
**ATP-dependent 6-phosphofructokinase, platelet type**	Q01813	PFKP PFKF	85,596	784	canonical glycolysis [GO:0061621]	6-phosphofructokinase activity [GO:0003872]; AMP binding [GO:0016208]; ATP binding [GO:0005524];
**Transitional endoplasmic reticulum ATPase**	P55072	VCP	89,322	806	NADH metabolic process [GO:0006734] positive regulation of ATP biosynthetic process [GO:2001171] positive regulation of canonical Wnt signaling pathway [GO:0090263]	ADP binding [GO:0043531]; ATPase activity [GO:0016887]; ATP binding [GO:0005524]; BAT3 complex binding [GO:1904288]; deubiquitinase activator activity [GO:0035800];
**Tripeptidyl-peptidase 2**	P29144	TPP2	138,350	1249	protein polyubiquitination [GO:0000209]	endopeptidase activity [GO:0004175]; identical protein binding [GO:0042802]; serine-type endopeptidase activity [GO:0004252]; tripeptidyl-peptidase activity [GO:0008240]

**Isoleucine--tRNA ligase, cytoplasmic**	P41252	IARS1 IARS	144,498	1262	isoleucyl-tRNA aminoacylation [GO:0006428]	aminoacyl-tRNA editing activity [GO:0002161]; ATP binding [GO:0005524]; GTPase binding [GO:0051020]; isoleucine-tRNA ligase activity [GO:0004822]; tRNA binding [GO:0000049]
**Trypsin-3**	P35030	PRSS3 PRSS4 TRY3 TRY4	32,529	304	cobalamin metabolic process [GO:0009235]	calcium ion binding [GO:0005509]; serine-type endopeptidase activity [GO:0004252]; serine-type peptidase activity [GO:0008236]
**Glutamine--tRNA ligase**	P47897	QARS1 QARS	87,799	775	brain development [GO:0007420]	ATP binding [GO:0005524]; glutamine-tRNA ligase activity [GO:0004819]; protein kinase binding [GO:0019901]; protein kinase inhibitor activity [GO:0004860]
**Trifunctional enzyme subunit alpha, mitochondrial**	P40939	HADHA HADH	83,000	763	fatty acid beta-oxidation [GO:0006635] response to drug [GO:0042493] response to insulin [GO:0032868]	3-hydroxyacyl-CoA dehydrogenase activity [GO:0003857]; acetyl-CoA C-acetyltransferase activity [GO:0003985]; enoyl-CoA hydratase activity [GO:0004300]; fatty-acyl-CoA binding [GO:0000062]; long-chain-3-hydroxyacyl-CoA
**ATP-dependent RNA helicase DDX1**	Q92499	DDX1	82,432	740	multicellular organism development [GO:0007275] nucleic acid phosphodiester bond hydrolysis [GO:0090305]	ATP binding [GO:0005524]; chromatin binding [GO:0003682]; DNA/RNA helicase activity [GO:0033677];
**Xaa-Pro aminopeptidase 1**	Q9NQW7	XPNPEP1 XPNPEPL XPNPEPL1	69,918	623	proteolysis [GO:0006508]	aminopeptidase activity [GO:0004177]; manganese ion binding [GO:0030145]; metalloaminopeptidase activity [GO:0070006]; protein homodimerization activity [GO:0042803]
**Caveolae-associated protein 1**	Q6NZI2	CAVIN1 PTRF FKSG13	43,476	390	positive regulation of cell motility [GO:2000147] protein secretion [GO:0009306]	identical protein binding [GO:0042802]; RNA binding [GO:0003723]; rRNA primary transcript binding [GO:0042134]
